# Disseminated Bacillus Calmette–Guérin (BCG) infection and acute exacerbation of interstitial pneumonitis: an autopsy case report and literature review

**DOI:** 10.1186/s12879-020-05396-7

**Published:** 2020-09-29

**Authors:** Gen Shimizu, Ryota Amano, Itaru Nakamura, Akane Wada, Masanobu Kitagawa, Shuta Toru

**Affiliations:** 1 Department of Neurology, Nitobe Memorial Nakano General Hospital, 4-59-16 Chuo Nakano-ku, Tokyo, 164-8607 Japan; 2 Department of Infection Prevention and Control, Tokyo Medical University, 6-7-1 Nishi-shinjuku, Shinjuku-ku, Tokyo, 160-0023 Japan; 3 Department of Oral Pathology, Tokyo Medical and Dental University, 1-5-45 Yushima, Bunkyo-ku, Tokyo, Japan; 4 Department of Comprehensive Pathology, Tokyo Medical and Dental University, 1-5-45 Yushima, Bunkyo-ku, Tokyo, Japan

**Keywords:** Bacillus Calmette–Guérin, Interstitial pneumonitis, Hepatosplenomegaly, Myelosuppression, Exanthema, BCG infection, *Mycobacterium bovis*

## Abstract

**Background:**

Intravesical administration of Bacillus Calmette–Guérin (BCG) has proven useful for treatment and prevention of recurrence of superficial bladder cancer and in situ carcinoma. However, fatal side effects such as disseminated infections may occur. Early diagnosis and accurate therapy for interstitial pneumonitis (IP) are important because exacerbation of IP triggered by infections is the major cause of death. Although some fatality reports have suggested newly appeared IP after intravesical BCG treatment, to our knowledge, there are no reports which have demonstrated acute exacerbation of existing IP. Moreover, autopsy is lacking in previous reports. We report the case of a patient with fatal IP exacerbation after BCG instillation and the pathological findings of the autopsy.

**Case presentation:**

A 77-year-old man with a medical history of IP was referred to our hospital because of fever and malaise. He had received an intravesical injection of BCG 1 day before the admission. His fever reduced after the use of antituberculosis drugs, so he was discharged home. He was referred to our hospital again because of a high fever 7 days after discharge. On hospitalisation, he showed high fever and systemic exanthema. Hepatosplenomegaly and myelosuppression were also observed. Biopsies revealed multiple epithelioid cell granulomas with Langhans giant cells of the liver and bone marrow. Biopsy DNA analyses of *Mycobacterium bovis* in the bone marrow, sputum, and blood were negative. His oxygen demand worsened drastically, and the ground-glass shadow expanded on the computed tomography scan. He was diagnosed with acute exacerbation of existing IP. We recommenced the antituberculosis drugs with steroid pulse therapy, but he died on day 35 because of respiratory failure. The autopsy revealed a diffuse appearance of multiple epithelioid cell granulomas with Langhans giant cells in multiple organs, although BCG was not evident.

**Conclusions:**

We report the first case of acute exacerbation of chronic IP by BCG infection. This is also the first case of autopsy of a patient with acute exacerbation of existing IP induced by intravesical BCG treatment. Whether the trigger of acute IP exacerbation is infection or hypersensitivity to BCG is still controversial, because pathological evidence confirming BCG infection is lacking. Physicians who administer BCG against bladder cancer should be vigilant for acute exacerbation of IP.

## Background

Intravesical administration of Bacillus Calmette–Guérin (BCG) has proven useful for treatment and prevention of recurrence of superficial bladder cancer and in situ carcinoma [1]. Although most patients have improved clinical outcomes without fatal complications, both local and systemic complications may occur following instillation [2]. The underlying mechanisms of such complications are not fully understood. However, hypersensitivity reactions and mycobacterial infection are at least two possibilities [3, 4]. Interstitial pneumonitis (IP) is reported to occur in 0.7% of patients treated with intravesical administration of BCG [5]. However, to our knowledge, there have been no reports of patients with acute exacerbation of existing IP. We report the case of a patient with fatal IP after intravesical BCG treatment and the pathological findings of the autopsy.

## Case presentation

A 77-year-old man with fever and malaise was referred to our hospital. He had undergone transurethral resection of a bladder tumour on the right wall 1 month before admission. Because the pathological diagnosis was malignant cancer, he had received an 40 mg of intravesical injection of BCG 1 day before the admission for the first time. His medical history included hypertension, diabetes, dyslipidaemia and subclinical IP, which had been diagnosed 5 months ago at a medical check-up. He had smoked around 20 cigarettes a day until 14 years ago.

**Fig. 1:**
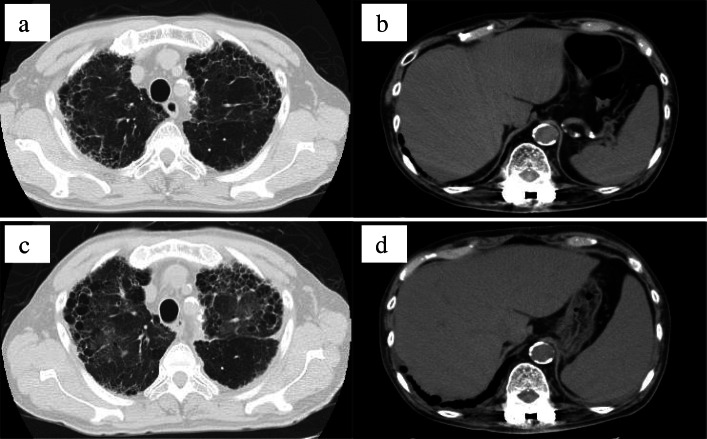
Computed tomography (CT) scan of interstitial pneumonia (IP) and hepatosplenomegaly. IP with reduced activity (**a**), and normal-size liver and spleen on day 1 (**b**). Acute exacerbation of IP with expanded ground-glass shadow in both lung areas on day 20 (**c**). Aggressive hepatosplenomegaly on day 20 (**d**)

On the first admission, his vital signs were as follows: body temperature 38.6 °C, heart rate 102 beats/min, and blood pressure 138/87 mmHg. His oxygen saturation was 94% on room air. Physical examination revealed fine crackles in the bilateral chest. Laboratory examinations revealed slightly high serum levels of hepatobiliary enzymes (AST 84 IU/L, ALT 39 IU/L, γ-GTP 116 IU/L, LD 268 IU/L, T-Bil 2.00 mg/dL) and C − reactive protein (CRP; 6.7 mg/dL). The white blood cell (WBC) counts were within the normal ranges. Sputum culture, urinary culture and two sets of blood cultures were negative for bacterial, mycobacterial, and fungal cultures. Mycobacterial cultures were performed by using BD BACTEC MGIT960® (Becton, Dickinson and company, New Jersey). A chest computed tomography (CT) scan revealed bilateral ground-glass shadow, unchanged compared to 5 months prior (Fig. 1a).

Because his fever occurred soon after the intravesical BCG injection, infection with BCG was suspected. The patient received antituberculosis drugs including rifampicin (RFP), isoniazid (INH) and ethambutol (EB). His fever, serum levels of hepatobiliary enzymes, and CRP immediately decreased to normal ranges within 4 days. He was discharged home on the sixth day with continuing antituberculosis drugs.

**Fig. 2:**
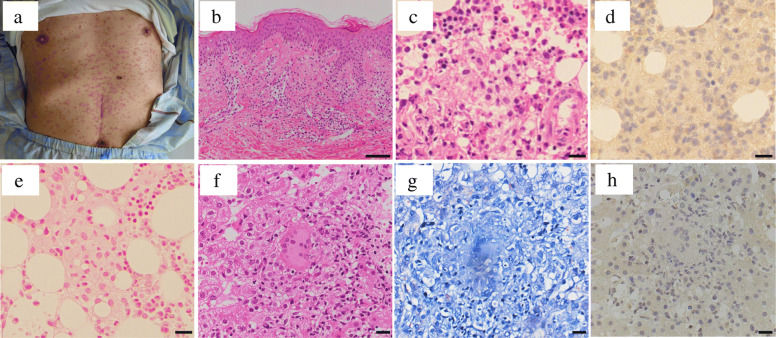
Systemic exanthema with rice grain–sized red papules on day 14. Biopsy of the skin revealed drug eruption (**a**, **b**). Epithelioid cell granuloma on bone marrow biopsy (**c**–**e**) and on liver biopsy (**f**–**h**). Bar in B = 200 μm. Bar in C–H = 20 μm. [B: haematoxylin–eosin (HE), × 40; C, F: HE, × 400; D, H: BCG(DAKO)-IHC, × 400; E: Berlin blue, × 400; G: Ziehl–Neelsen, × 400]

However, he was referred to our hospital again because of a high fever 5 days after discharge. On readmission, his vital signs were as follows: body temperature 39.9 °C, heart rate 115 beats/min, and blood pressure 166/74 mmHg. His oxygen saturation was 93% on room air. Fine crackles on the bilateral chest remained. He also showed rice grain–sized red papules on broad areas of his face, and red bean–sized erythema with pruritus and infiltration over his trunk and limbs (Fig. 2a). No other abnormal findings were noted on physical examination.

Laboratory examinations revealed high serum levels of hepatobiliary enzymes (AST 57 IU/L, ALT 41 IU/L, γ-GTP 552 IU/L, LD 390 IU/L, ALP 653 IU/L, T-Bil 1.89 mg/dL) and CRP (5.2 mg/dL). Other blood examinations, including WBC, were within the normal ranges. T-SPOT.TB (a type of interferon-gamma release assay) was negative. We took sputum culture, urinary culture and two sets of blood cultures again. However, they were negative for bacterial, mycobacterial, and fungal cultures.

A chest CT scan revealed bilateral ground-glass shadow, unchanged since the last hospitalisation. We could not detect the inflammatory focus by CT scan, so we presumed three possibilities for his fever: 1. initial aggravation of the BCG treatment: an allergic reaction against antigens of dead mycobacterium, 2. exacerbation of infection: inadequate treatment, 3. antituberculosis drug–induced fever. To make the correct diagnosis, we stopped the antituberculosis drugs. Skin biopsy of the exanthema revealed a drug eruption (Fig. 2b). The exanthema had regressed on the 20th day of hospitalisation after discontinuing antituberculosis drugs.

On day 20 from the first admission, a contrast-enhanced CT scan revealed hepatosplenomegaly (Fig. 1d). Liver biopsy revealed multiple epithelioid cell granulomas with Langhans giant cells (Fig. 2f). Caseous necrosis was not observed. Furthermore, *Mycobacterium* was not detected on Ziehl–Neelsen stain (Fig. 2g) or the rabbit polyclonal anti-BCG antibody (Dako)–based immunohistochemistry (IHC; Fig. 2h).

Leukopenia gradually proceeded after hospitalisation (day 12: 3900/μL, day 13: 5410/μL, day 18: 2110/μL). The blood level of haemoglobin decreased following the leukopenia. Bone marrow biopsy revealed multiple epithelioid granulomas (Fig. 2c). In the epithelioid cell granulomas, neither caseous necrosis nor *Mycobacterium* was detected with Ziehl–Neelsen stain and anti-BCG IHC (Fig. 2d). Berlin blue stain was negative (Fig. 2e). The bone marrow infection of BCG was suspected to be responsible for the leukoerythropenia. However, bone marrow, sputum and blood PCR for *Mycobacterium* (COBAS® TaqMan® MTB, Roche Diagnostics, Tokyo) were negative.

On day 20, the follow-up CT scan demonstrated a new ground-glass shadow on the bilateral upper lobe (Fig. 1c). The patient’s oxygen demand gradually worsened at this point (oxygen saturation was 94% on 2 L/min of oxygen). On day 27, his oxygen demand worsened drastically, and the ground-glass shadow had expanded on the CT scan. Moreover, the serum levels of KL-6 (1020 U/mL) and SP-D (330 ng/mL) were high, suggesting acute exacerbation of IP. The serum level of adenosine deaminase (ADA) was also high (183.2 U/L). From the high levels of ADA, KL-6, SP-D and the pathological findings of the biopsies, we diagnosed an acute exacerbation of IP associated with *Mycobacterium bovis* infection or hypersensitivity. We restarted antituberculosis drugs immediately. Because the antituberculosis drugs were suspected of causing drug eruption, we changed RFP to rifabutin (RFB). Furthermore, we used INH at a desensitisation amount and gradually increased. For IP, high-dose methylprednisolone (500 mg/day × 3 days intravenously) was also commenced. Prednisolone (50 mg/day [1 mg/kg] orally) was administered after the high-dose methylprednisolone.

Although no allergic symptoms occurred, including exanthema, the patient’s respiratory condition gradually worsened, and he died on day 35. The results of a drug-induced lymphocyte stimulation test on day 20 were negative for RFP, INH and EB.

### An autopsy was performed, with the following pathological findings

**Fig. 3:**
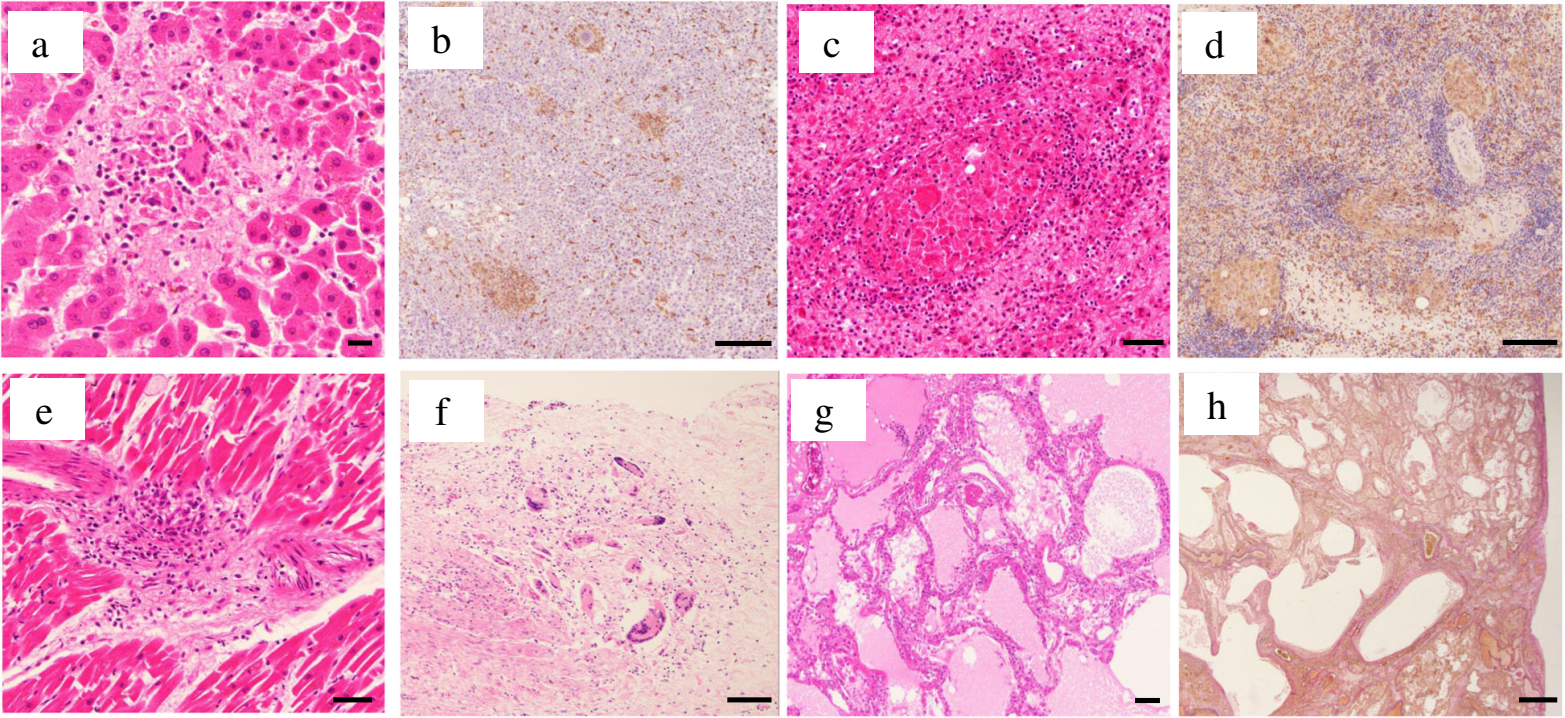
Microscopy of the autopsy showed epithelioid cell granulomas on the liver (**a**, **b**), spleen (**c**, **d**) and heart (**e**), and Langhans giant cells on the urinary bladder (**f**). Microscopy of the lung specimen revealed pulmonary oedema with hyaline membranes (**g**) and patchy paraseptal fibrosis with remodelling of the lung architecture (**h**), suggesting acute exacerbation of interstitial pneumonitis. Bar in A = 20 μm. Bar in B, D, G = 200 μm. Bar in C, E, F = 50 μm. Bar in H = 1 mm. [A: haematoxylin–eosin (HE), × 400; B, D: CD68(Kp-1;DAKO)-IHC, × 100; C, E, F: HE, × 200; G: HE, × 40; H: EvG, × 12.5]

On haematoxylin–eosin (HE) staining and anti-CD68 (Kp-1, DAKO) IHC, a diffuse appearance of multiple epithelioid cell granulomas with Langhans giant cells was observed in the liver (Fig. 3a, b), spleen (Fig. 3c, d) and bone marrow [1]. Small numbers of epithelioid cell granulomas were observed in the heart (Fig. 3e). They were variously degenerated through post-mortem changes. In the wall of the urinary bladder, aggregations of Langhans giant cells were observed, but epithelioid cell granuloma was not detected (Fig. 3f). Anti-BCG and anti-lipoarabinomannan antibody–based IHC were negative in all of the above. We could not detect *Mycobacterium* histopathologically. No remaining carcinoma cells were found in the urinary bladder.

The left lung weighed 710 g and the right lung weighed 880 g [2]. Patchy interstitial fibrosis with remodelling of the lung architecture resulting in formation of cystic spaces and honeycombing was observed in a subpleural and paraseptal distribution, alternating with areas of structurally preserved lung (Fig. 3h). Mild interstitial lymphocyte infiltration was seen. These observations were compatible with the pattern of chronically developed usual interstitial pneumonitis (UIP). The structurally preserved area showed diffuse involvement by alveolar oedema with hyaline membranes in the upper lobes (Fig. 3g), and by intra-alveolar plugs of organising connective tissue mainly in the lower lobes. These findings suggested diffuse alveolar damage from the exudative phase to the organising phase occurring over 1–3 weeks. From the observations of diffuse alveolar damage with UIP, we diagnosed acute exacerbation of UIP. We also performed PCR for *Mycobacterium* on liver, spleen, lung, ileum, stomach, and cerebrospinal fluid on autopsy specimens, however, the results were all negative.

## Discussion and conclusion

We report a patient with mortal acute exacerbation of IP after intravesical BCG treatment. Pneumonitis is a rare complication of intravesical BCG treatment that occurs in less than 0.7% of patients following the repeated administration of BCG [5]. Some reports IP following BCG use. However, no reports have involved patients with chronic IP. Moreover, to our knowledge, this is the first case with anatomical pathology performed after BCG instillation.

Pathological findings revealed granuloma with Langhans giant cells in the liver, bone marrow, spleen and heart (Fig. 3a–e). From the episode of intravesical BCG treatment and the distribution of epithelioid cell granulomas, it is reasonable that the granuloma formation was a result of BCG dissemination, with or without hypersensitivity to its adjuvant, at least originally. If only the hypersensitivity to BCG had induced his clinical symptoms, the granulomas would have been found in the other organs. So, we suspect that, at least once, the BCG dissemination had occurred. In this case, we initially diagnosed BCG infection because the patient’s fever reduced only with administration of antituberculosis drugs. However, the examinations for *Mycobacterium* infection, including stain for acid-fast bacilli, culture, and PCR-based assay were all negative. Pérez et al. reported that microbiological-based diagnosis for BCG infection was positive in only 118 of 246 patients [4]. This suggests that all microbiological examinations being negative does not exclude the possibility of BCG infection.

Besides the acute exacerbation of IP, myelosuppression and hepatosplenomegaly were observed in this patient. Myelosuppression is reported in 0.1% of patients after BCG treatment [5]. It appears in tuberculosis when the bone marrow is involved in the infection. Cho et al. reported a patient who showed myelosuppression after intravesical BCG treatment. In that report, granulomas were found in the bone marrow biopsy [6]. Some reports have described miliary tuberculosis inducing myelosuppression. In those reports, antituberculosis drugs or other haematological disease complicated with tuberculosis have been suspected as one cause of myelosuppression [7]. In the present case, anti-BCG antibody–based IHC detected no *Mycobacterium* in the bone marrow biopsy, so whether the myelosuppression was induced by the infection or hypersensitivity is uncertain.

Hepatitis has been reported in 0.7% of patients after intravesical BCG treatment [5]. The mechanism of granulomatous hepatitis (as well as IP) after intravesical BCG treatment is not clear, but hypersensitivity and BCG infection have been proposed.

Since the mechanisms are still unclear, the treatment of IP following intravesical BCG treatment has not been established. Some reports have recommended using steroids because of the possibility of hypersensitivity [8, 9]. In particular, Israel-Biet et al. described three cases of IP related to intravesical BCG treatment [9]. They reported that the pulmonary complications induced by intravesical BCG therapy are rarely related to infection, because of negative bronchoalveolar lavage (BAL) cultures for *mycobacterium* and the increased population of activated lymphocytes highly sensitised to purified protein derivative on BAL. Moreover, one of the three patients fully recovered with corticosteroids alone, without antituberculosis drugs [9]. However, other reports have recommended combination therapy with antituberculosis drugs and steroids because of the possibility of infection [5]. Table 1 shows the previously reported cases of IP secondary to intravesical BCG treatment. Except for one case, all patients were treated with combination therapy using antituberculosis drugs and steroids (Table 1). The remaining case (case 13 in Table 1) received tacrolimus (TAC) and everolimus (EVL) for kidney transplantation; therefore, all patients received immunosuppression therapies. Nine out of sixteen cases underwent steroid pulse therapy and six recovered. All of the seven cases without steroid pulse therapy recovered. Although not enough cases exist for definitive conclusions, the use of steroid pulse therapy might have no effect on prognosis. In this case, we tried steroid pulse therapy with antituberculosis drugs; however, the patient’s IP did not improve. Kitani et al. reported high mortality in IP secondary to intravesical BCG treatment, at 26.9% [22]. Besides the high mortality, acute exacerbation of past IP might be a reason for the treatment resistance in this case.

**Table 1:** Published cases of interstitial pneumonitis secondary to intravesical BCG

Reference	Case	Age	BCC treatment before onset	Time from final BCC treatment to onset	Time from final BCC treatment to dyspnea	Antituberculosis	Steroid	Other symptoms	Prognosis
Diner [10]	1	81	6 weeks + 4 weeks of maintenance	1 day	2 weeks	INH + RFP + EB	PSL 30 mg	Fever	Alive
Cho [6]	2	76	Not specified	7 months	7 months	INH + RFP + EB	Pulse	Fever, pancytopenia	Alive
Lyons [11]	3	78	4 weeks	6 h	3 weeks	INH + RFP	PSL 40 mg	Fever, hepatitis	Alive
Carrasco [12]	4	73	8 times	10 days	Not specified	Not specified	mPSL 40 mg	Fever, hepatitis	Alive
Um [3]	5	60	3 times	3 weeks	3 weeks	Not specified	cortisteroid	Fever, hepatitis	Alive
Naoki [13]	6	61	3 times	1 week	3 weeks	INH + RFP + EB	Pulse	Fever, hepatitis	Alive
Nitta [14]	7	60	6 times + maintenance BCG	1 day	2 weeks	INH + RFP + EB	Pulse	Fever	Alive
Horinaga [15]	8	61	3 times	Not specified	Not specified	INH + RFP + SM	Pulse	Fever, hepatitis	Alive
Uetsuki [16]	9	49	8 times+ 5 times	Not specified	Not specified	INH + RFP	Pulse	Fever, hepatitis	Alive
Yamamoto [17]	10	81	5 times	1 week	2 weeks	INH + RFP	Pulse	Fever	Died
Davis [18]	11	79	7 times	1 week	12 days	INH + RFP + EB	PSL 60 mg	Fever	Alive
Tobiume [19]	12	86	3 times	6 days	6 days	INH + RFP	Pulse	Fever	Alive
Caravaca [20]	13	81	1 time	30 days	Not specified	INH + RFP + EB	Not used (TAC + EVL)	Fever	Alive
Azumi [21]	14	73	6 times + 2 times	Not specified	50 days after admission	INH + RFP + EB	Pulse	Fever, nephromegaly	Died
Kitani [22]	15	85	3 times	The same day	The same day	Not used	PSL 250 mg	Fever	Alive
Kazuaki [23]	16	72	6 times	1 weeks	2 weeks	Not used	Pulse	nausea	Died

Intravesical BCG treatment has proven effectiveness against urothelial cancer, but its rare side effects can be fatal. For patients receiving intravesical BCG treatment, close observation for respiratory condition, myelosuppression and hepatitis is necessary for early therapeutic intervention. Early diagnosis and treatment can improve the clinical outcomes of the patients with IP secondary to intravesical BCG treatment.

## Data Availability

All data related to this case report are contained within the article.

## Abbreviations

ADA: Adenosine deaminase
ALT: Alanine aminotransferase
AST: Aspartate transaminase
BAL: Bronchoalveolar lavage
BCG: Bacillus Calmette–Guérin
CD: Cluster differentiation
CRP: C-reactive protein
CT: Computed tomography
DAD: Diffuse alveolar damage
DNA: Deoxyribonucleic acid
EB: Ethambutol
ɤ-GTP: Gamma-glutamyltranspeptidase
EVL: Everolimus
HE: Haematoxylin–eosin
IHC: Immunohistochemistry
INH: Isoniazid
IP: Interstitial pneumonitis
KL-6: Krebs von den Lungen-6
SP-D: Surfactant protein-D
LD: Lactate dehydrogenase
PCR: Polymerase chain reaction
PPD: Purified protein derivative
RFB: Rifabutin
RFP: Rifampicin
TAC: Tacrolimus
T-Bil: Total-bilirubin
TUR-BT: Transurethral resection of the bladder tumour
UIP: Usual interstitial pneumonitis
WBC: White blood cell
